# Antitumoral activity of the mithralog EC-8042 in triple negative breast cancer linked to cell cycle arrest in G2

**DOI:** 10.18632/oncotarget.5942

**Published:** 2015-09-30

**Authors:** Atanasio Pandiella, Francisco Morís, Alberto Ocaña, Luz-Elena Núñez, Juan C. Montero

**Affiliations:** ^1^ Instituto de Biología Molecular y Celular del Cáncer, CSIC-Universidad de Salamanca, Salamanca, Spain; ^2^ EntreChem SL, Oviedo, Spain; ^3^ Translational Research Unit, Complejo Hospitalario Universitario de Albacete, Albacete, Spain

**Keywords:** mithralogs, cell cycle, triple negative breast cancer, EC-8042

## Abstract

Triple negative breast cancer (TNBC) is an aggressive form of breast cancer. Despite response to chemotherapy, relapses are frequent and resistance to available treatments is often observed in the metastatic setting. Therefore, identification of new therapeutic strategies is required. Here we have investigated the effect of the mithramycin analog EC-8042 (demycarosil-3D-β-D-digitoxosyl mithramycin SK) on TNBC. The drug caused a dose-dependent inhibition of proliferation of a set of TNBC cell lines *in vitro*, and decreased tumor growth in mice xenografted with TNBC cells. Mechanistically, EC-8042 caused an arrest in the G2 phase of the cell cycle, coincident with an increase in pCDK1 and Wee1 levels in cells treated with the drug. In addition, prolonged treatment with the drug also causes apoptosis, mainly through caspase-independent routes. Importantly, EC-8042 synergized with drugs commonly used in the therapy of TNBC *in vitro*, and potentiated the antitumoral effect of docetaxel *in vivo*. Together, these data suggest that the mithralog EC-8042 exerts an antitumoral action on TNBC cells and reinforces the action of standard of care drugs used in the therapy of this disease. These characteristics, together with a better toxicology profile of EC-8042 with respect to mithramycin, open the possibility of its clinical evaluation.

## INTRODUCTION

Triple negative breast cancers (TNBCs) comprise 15% of all breast tumors, and are defined at immunohistochemical level as tumors lacking detectable expression of hormone receptors, and no HER2 gene amplification [1–3]. Treatment of TNBC is based on chemotherapy, effective in these tumors because of their rapid proliferation rates and frequent derangements in DNA repair mechanisms [4]. Unfortunately, relapses are frequent, and resistance to the chemotherapeutic agents is often encountered in the metastatic setting [4–6]. These facts, together with the relatively poor knowledge of the driver molecular alterations present in TNBC have stimulated research to identify aberrant signaling networks that may be pharmacologically attacked [4].

A recent RNAi screen in search for agents that enhance paclitaxel activity in TNBC identified mithramycin as an agent able to sensitize TNBC cells to the antitumoral effect of taxanes [7]. Mithramycin is a reversible DNA binding antitumoral antibiotic approved since 1970 by the FDA, although severe side effects have limited its use in the clinic. Recently, promising *in vitro* and *in vivo* activity linked to specific modes of action [8, 9] have triggered its clinical evaluation in Ewing sarcoma, lung, esophagus and other thoracic malignancies [10]. The mode of action of mithramycin and several of its analogs involves a non-covalent interaction with GC-rich DNA regions located at the minor groove of DNA [11–13]. Several studies have pointed out that the basis for the antitumor properties of mithramycin and other analogs tested to date is the inhibition of replication and transcription processes during macromolecular biosynthesis by interacting with GC-rich nucleotide sequences, especially the site of union of Sp1 transcription factor [14–17]. As a consequence, proteins whose expression is affected by this drug include various protooncogenes, proteins involved in angiogenesis or antiapoptotic processes, p53-mediated transcriptional responses, as well as multidrug resistant gene 1 (MDR-1) [18]. All these facts have fostered interest in the development of mithramycin analogs (mithralogs) with improved properties, focusing on those with lower toxicity, thus having better clinical chances than the wild type natural product [19]. Demycarosyl-3D-β-D-digitoxosyl-mithramycin SK (EC-8042) is a mithramycin analog generated by combinatorial biosynthesis currently under development as antitumor agent. EC-8042 is a lead molecule in the quest for mithralogs with improved therapeutic window, since it is 10 times less toxic than mithramycin *in vivo*, while it is active both *in vitro* and in cancer xenograft models [20]. Moreover, recent gene expression array data have confirmed not only the role of Sp1 inhibition in the mechanism of action of EC-8042, but also other signaling networks relevant in cancer [21].

These precedents led us to explore the potential antitumoral action of EC-8042 against TNBC. Here we show that EC-8042 is active against a panel of TNBC cell lines and exerts antitumoral properties in TNBC cells xenografted in mice. Moreover, the drug augmented the action of compounds used clinically in the treatment of TNBC. Studies on the mechanism of action demonstrated that the drug exerted its action by a complex mechanism that involved cell cycle and proapoptotic effects. Together, the data suggest that EC-8042 could be considered for further development with the aim of being incorporated to the armamentarium used in the therapy of the disease.

## RESULTS

### Effect of EC-8042 on TNBC cell lines

**Figure 1:**
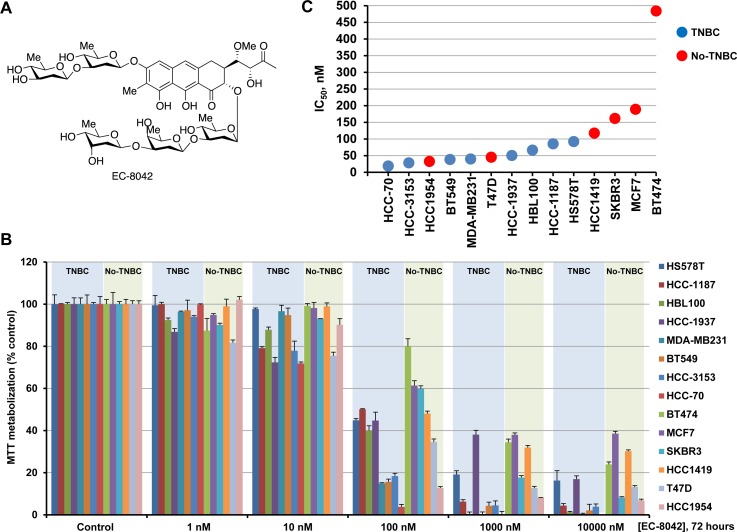
Action of EC-8042 in breast cancer cell lines **A.** Structure of EC-8042. **B.** Dose-response analyses of the effect of EC-8042 on the different breast cancer cell lines. Cells were treated with EC-8042 for 72 hours at the indicated doses. The data are plotted as the percentage of MTT metabolization with respect to control, vehicle-treated cells. Results are shown as the mean ± SD of quadruplicates of an experiment repeated three times. **C.** IC_50_ values obtained in the different breast cancer cell lines after 72 hours of incubation with EC-8042.

The structure of demycarosyl-3D-β-D-digitoxosyl-mithramycin SK (EC-8042) is shown in Figure 1A. To analyze the potential antineoplastic effect of the drug on TNBC, we evaluated its action on a panel of eight representative TNBC cell lines using MTT metabolization as readout of the effect of the drug on cell number. In addition, we also explored the action of EC-8042 on six additional breast cancer cell lines which belong to other subtypes of breast cancer (two hormone receptor positive, and four HER2+). Cells were plated and treated for 48 (Figure S1) or 72 hours (Figure 1B) with different doses of EC-8042. Treatment with EC-8042 decreased MTT metabolization of all the cell lines studied in a dose-dependent manner. At 72 hours of treatment, the drug IC_50_ values for all the TNBC cell lines were below 100 nM (Figure 1C), indicating that EC-8042 was a potent inhibitor of the MTT metabolization in these cells. The IC_50_ values observed for EC-8042 were in general higher in non-TNBC breast cancer cell lines than in the case of the TNBC cell lines, indicating that EC-8042 could be better suited to be used in TNBC than in other breast cancer subtypes.

### EC-8042 provokes cell cycle arrest in G2

**Figure 2:**
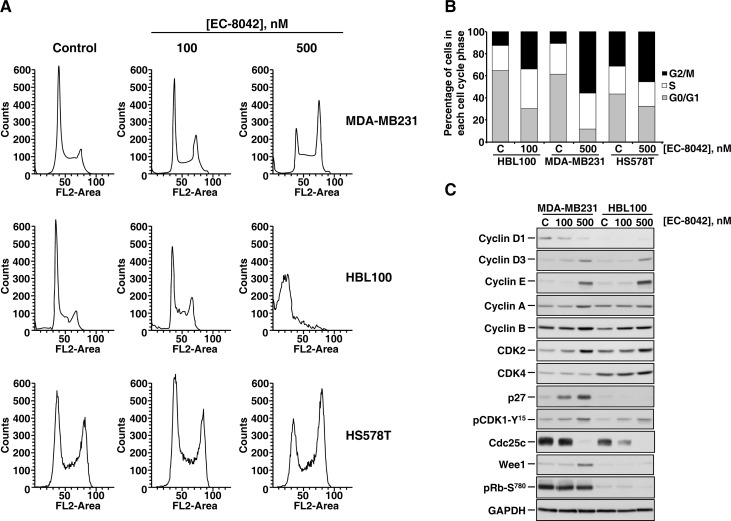
Effect of EC-8042 on the cell cycle **A.** Propidium iodide stainings of MDA-MB231, HBL100, and HS578T cells treated for 48 hours with 100 or 500 nM of EC-8042. **B.** Quantitative analyses of the action of EC-8042 on the distribution of the different cell cycle phases in the TNBC cell lines indicated. Cells were treated with the indicated concentrations of EC-8042 for 48 hours. **C.** Biochemical analyses of proteins involved in cell cycle progression. Cells were treated for 24 hours with the indicated concentrations of EC-8042, and lysates prepared. Analyses of the amounts of the different proteins studied were performed by Western blotting. GAPDH was used as a loading control.

The decrease in MTT metabolization caused by EC-8042 could be caused by reduction in cell proliferation, increased cell death or a combination of both. To further analyze the antiproliferative mechanism of action of EC-8042 DNA stainings of MDA-MB231, HS578T and HBL100 cells were performed. Addition of EC-8042 to these cells increased the magnitude of the peak of the histogram corresponding to cells in the G2 or M phases (Figure 2A and 2B). A concomitant decrease of cells in the peak corresponding to the G1 phase was observed. In HBL100 cells, treatment with the drug also caused an increase in cells accumulating in G2/M at 100 nM. At 500 nM, the drug provoked accumulation of cells in the subG1 region of the histogram, suggestive of induction of cell death. This subG1 accumulation of cells treated at 500 nM of the compound was also observed in MDA-MB231, although to a much lesser extent.

The biochemical effects of EC-8042 on cell cycle regulatory proteins were evaluated in continuously growing cultures of MDA-MB231 and HBL100 cells. Western blotting analyses showed that treatment with EC-8042 caused a substantial decrease in the phosphatase cdc25C, together with an increase in Cyclin D3, Cyclin E, Cyclin A, Cyclin B, CDK2, and the phosphorylation of CDK1 at tyrosine 15. In MDA-MB231 cells the drug also increased the levels of p27 and Wee1 (Figure 2C). The above results confirmed that EC-8042 had an effect on proteins involved in the control of cell cycle progression.

**Figure 3:**
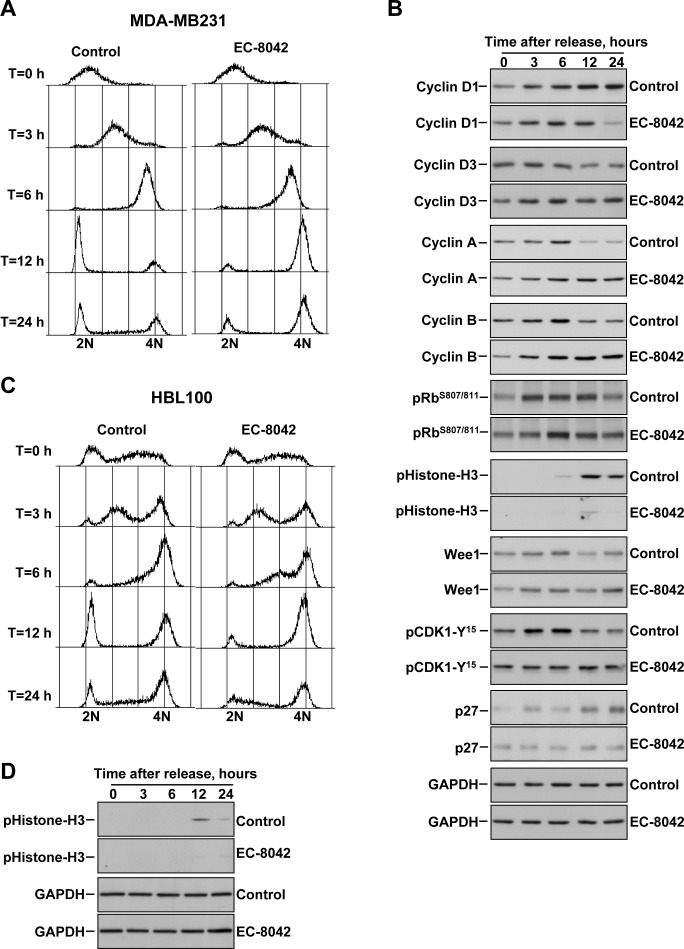
Effect of EC-8042 causes arrest of cells in the G2 cell cycle phase **A.** Propidium iodide stainings of MDA-MB231 cells synchronized in G1 by a double thymidine block, followed by release in the absence (Control) or presence of EC-8042 (500 nM). Chase times are shown at the left. **B.** Analyses of the levels of proteins involved in cell cycle progression at different chase times. Cells were synchronized in G1 by a double thymidine block, followed by release in the absence (Control) or presence of EC-8042 (500 nM). Western blotting was used to study the amounts of the different proteins analzyed. GAPDH was used as a loading control. **C.** Propidium iodide stainings of HBL100 cells synchronized in G1 by a double thymidine block, followed by release in the absence or presence of EC-8042 (500 nM). **D.** Analyses of the levels of pHistone-H3 and GAPDH in HBL100 cells released in the absence or presence of EC-8042.

To more accurately define the cell cycle effects of EC-8042 we performed cell synchronization experiments, followed by release of the cells in the absence or presence of EC-8042, and analyses of the cell cycle profiles as well as markers of cell cycle progression at different times after release. In MDA-MB231 cells, double thymidine treatment caused accumulation of cells in G1 (Figure 3A). At three and six hours after release the cells moved through S phase either in the absence or presence of EC-8042. At twelve hours after release, while most of the control cells have progressed through mitosis and accumulated at G1, cells chased in the presence of EC-8042 accumulated in the G2/M phase of the cell cycle. Biochemical analyses of Cyclins D1-D3 involved in G1 → S cell cycle progression indicated that EC-8042 did not substantially decrease their levels for up to 12 hours (Figure 3B). At 24 hours of treatment, a decrease in Cyclin D1 was observed in cells treated with EC-8042.

Sequential activation of CDK2 by Cyclin A and then CDK1 by Cyclins A and B are required to maintain Rb hyperphosphorylated to ensure cell cycle progression. Then, when cells enter mitosis, the amounts of both cyclins rapidly decrease [22]. Treatment with EC-8042 slightly delayed phosphorylation of Rb (Figure 3B). In control cells, levels of Cyclin A and Cyclin B rose, reaching a peak at 6 hours after release from the G1 blockade. At 12 hours after chase, the amounts of Cyclin A and B decreased with respect to the levels present in cells at 6 hours. In EC-8042-treated cells the levels of both cyclins progressively increased and remained elevated for up to 24 hours of treatment (Figure 3B).

Dephosphorylation of CDK1 at Y^15^, required for entry in mitosis, is controlled by the balance between the activity of the Wee1 kinase and the Cdc25c phosphatase. In control cells, pCDK1-Y^15^ levels decreased at 12 hours. In contrast, in cells treated with EC-8042 the levels of pCDK1-Y^15^ were sustained along the duration of the experiment (Figure 3B). Analyses of the phosphorylation of histone H3, which is used as a marker of cells in mitosis, indicated that cells treated with EC-8042 did not efficiently progressed into mitosis. Similar results were observed in HBL100 cells (Figure 3C and 3D).

With the purpose of evaluating whether a relationship existed among the levels of the proteins analyzed above and involved in the regulation of G2 → M transition and the sensitivity of the TNBC cell lines to EC-8042, the total amount of those proteins was measured and plotted against IC_50_ values (Figure S2). However, no correlation was found among the levels of Wee1, pCDK1-^Y15^ and IC_50_ values for EC-8042. While statistically significant correlation failed to be observed in the case of SP1 and Cdc25c, a trend towards resistance to the action of EC-8042 was found in cells expressing higher levels of these proteins. On the other side, higher levels of Cdc25a appeared to facilitate the action of the drug.

### EC-8042 induces cell death

**Figure 4:**
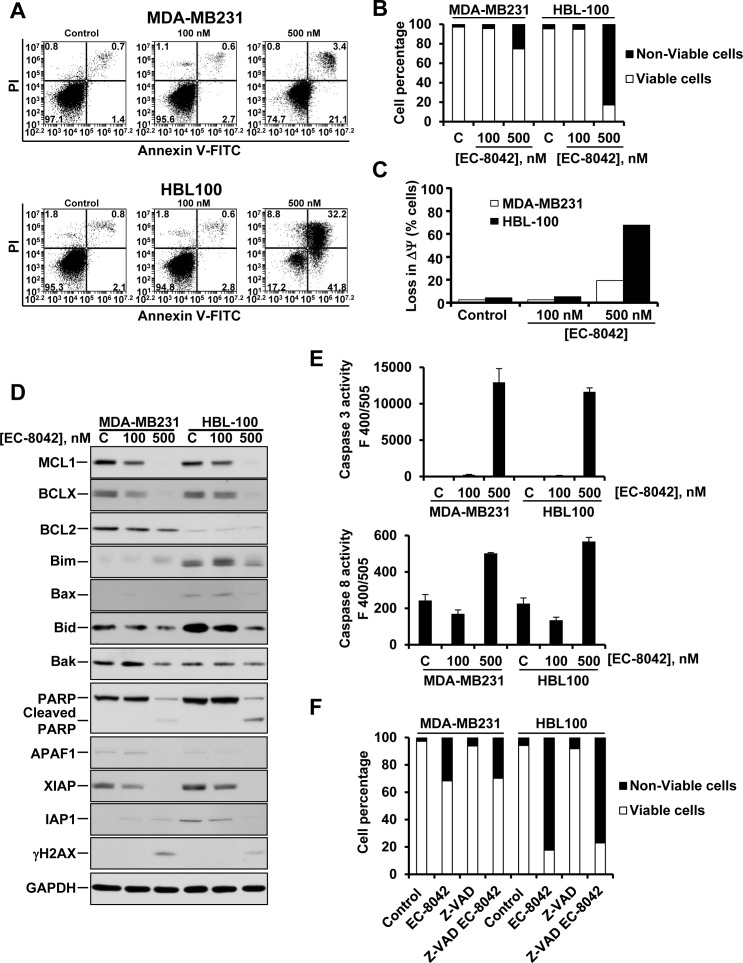
Effect of EC-8042 on apoptotic cell death **A.** Double Annexin V-propidium iodide stainings of MDA-MB231 and HBL100 cells treated for 48 hours with 100 or 500 nM EC-8042. **B.** Quantitative analyses of live/dead cells in cultures of MDA-MB231 and HBL100 cells treated for 48 hours with 100 or 500 nM EC-8042. **C.** Analyses of mitochondrial membrane potential in cells treated for 48 hours with the indicated concentrations of EC-8042. **D.** Effect of EC-8042 on the levels of several apoptosis-related proteins. MDA-MB231 and HBL100 cells were treated for 48 hours with 100 or 500 nM EC-8042, and protein levels analyzed by Western blotting. **E.** Analyses of caspase 3 and caspase 8 activities in MDA-MB231 and HBL100 cells were treated for 48 hours with 100 or 500 nM EC-8042. Data are presented as mean ± SD of duplicates, and plotted as the fluorescence values obtained. **F.** Effect of Z-VAD-FMK on the viability of cells treated with EC-8042. Cells were preincubated with 10 μM of Z-VAD-FMK for 30 minutes prior to addition of 500 nM EC-8042. Incubations were extended for 48 hours, after which Annexin V-Propidium iodide double stainings were performed.

The increase in cells in the subG1 phase of the cell cycle, especially in HBL100 cells treated with EC-8042 suggested that the drug stimulated cell death. As shown in Figure 4A, treatment with 500 nM of EC-8042 for 48 hours augmented Annexin V staining of HBL100 cells, and also increased Annexin V staining of MDA-MB231 cells, although to a much lesser extent (Figure 4A and 4B). Treatment with 500 nM EC-8042 resulted in loss of mitochondrial membrane potential in both HBL100 and MDA-MB231 cells (Figure 4C), although the effect was substantially higher in the HBL100 cells, in line with the increased apoptotic effect of EC-8042 on such cell line. Members of the BCL2 family of proteins are known to participate in the regulation of MOMP. We observed that MCL1 and BCLX levels were down regulated in both cell lines after treatment with EC-8042 for 48 hours (Figure 4D). We evaluated whether treatment with the drug caused an increase in caspase activity. Treatment with EC-8042 provoked an increase in caspase 3 and caspase 8 activities (Figure 4E). The drug also provoked a decrease in the caspase inhibitor XIAP. Moreover, an increase in PARP cleavage, indicative of activation of caspases, was observed in both MDA-MB231 and HBL100 cells (Figure 4D). To evaluate to which extent caspases were responsible of the cell death induced by EC-8042 cells were incubated with the pancaspase inhibitor Z-VAD-FMK before treatment with EC-8042. Addition of the caspase inhibitor did not inhibit EC-8042-induced cell death (Figure 4F) indicating that the effect of EC-8042 mainly occurred through caspase-independent processes.

### *In vivo* antitumoral effect of EC-8042

**Figure 5:**
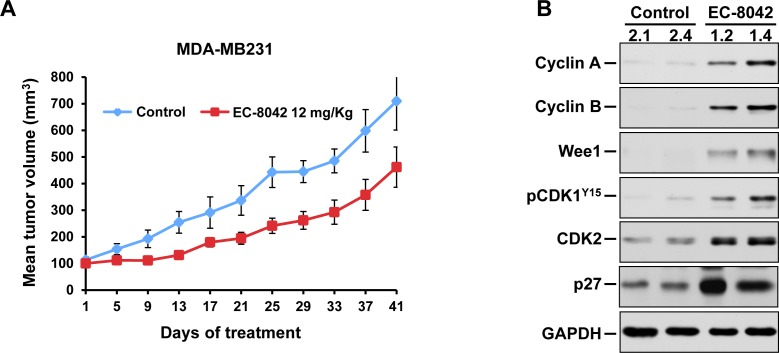
Antitumorigenic effect of EC-8042 *in vivo* **A.** MDA-MB231 cells were implanted in the mammary glands of female mice, and allowed to grow until tumors reached 100 mm^3^. Mice were randomized to receive vehicle (control) or 12 mg/Kg EC-8042 every 4 days. Data are plotted as mean tumor volumes ± SD of eight mice/group. **B.** Analysis of Cyclin A, Cyclin B, Wee1, pCDK1-Y^15^, CDK2, p27 and GAPDH in lysates from tumors obtained from control or EC-8042-treated mice 6 hours after the last treatment. The numbers refer to the animal from which the analyzed tumor was dissected.

The potential *in vivo* antitumoral effect of EC-8042 was next investigated. Mice injected with MDA-MB231 cells in the caudal mammary fat pad developed tumors within two weeks from the date of injection. Once tumors established and reached a mean volume of 100 mm^3^, mice were randomized to receive vehicle or EC-8042 (12 mg/kg), intravenously every four days. Pharmacokinetic measurements of EC-8042 concentration in the tumors at 6 hours after the last treatment indicated that the drug accumulated at doses higher that those deemed active *in vitro* (3.8 μg/g and 2.2 μg/g in two tumors from two different mice). Treatment with the drug exerted an inhibitory effect on tumor growth (Figure 5A). Body weight showed a slight decrease initially in animals treated with EC-8042, which recovered later (Figure S3).

The definition of the molecular consequences of the treatment of EC-8042 on cell cycle regulators reported above guided in performing a pharmacodynamic assessment of the action of the drug *in vivo*. Tumors from control or EC-8042-treated mice removed 6h after the last treatment showed that tumoral samples from mice treated with the drug had increased levels of Wee1, pCDK1-Y^15^, p27, Cyclin A and Cyclin B (Figure 5B).

### EC-8042 synergizes with standard of care drugs *in vitro* and *in vivo*

**Figure 6:**
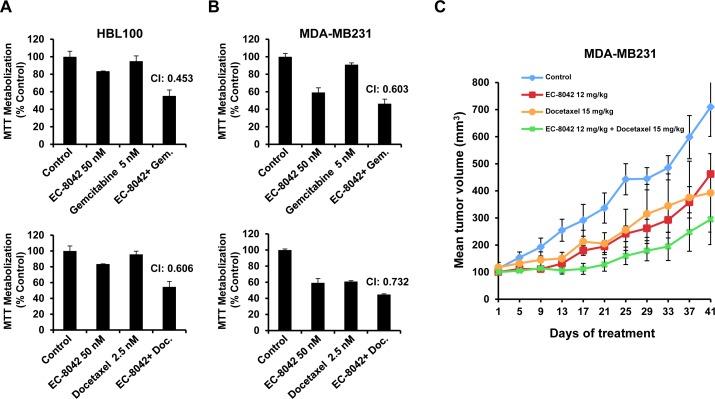
Synergy of EC-8042 with standard of care drugs HBL100 **A.** or MDA-MB231 **B.** cells were treated for 48 hours with a fixed ratio model of combinations of EC-8042, gemcitabine, or docetaxel. MTT metabolization analyses were performed and data analyzed using the Chou-Talalay algorithm. The results shown in the figure correspond to only a single dose combination example in which synergy was observed as indicated by CI values below 1. **C.** Analyses of the *in vivo* effect of drug combinations of tumor growth in nude mice implanted with MDA-MB231 cells. Tumors were allowed to grow to 100 mm^3^, and then mice were randomized to receive vehicle (control) or the indicated doses of the drugs. Data are plotted as mean tumor volumes ± SD of eight mice/group.

Antineoplastic treatments are often based on combinations of agents. To explore whether EC-8042 potentiated the action of drugs used in the therapy of TNBC, drug combination experiments were performed, and the results analyzed by the Chou and Talalay algorithm [23] which is used to determine whether a drug combination is synergistic, additive or antagonistic or has no effect. Synergy was observed with docetaxel and gemcitabine (Figure 6A and 6B), while the action of vinorelbine (Figure S4) or carboplatin were not clearly augmented by EC-8042. We also tested the possibility that EC-8042 favored the action of other drugs which may be useful in TNBC such as the PI3K/mTOR inhibitor BEZ235 or the tyrosine kinase inhibitor dasatinib. These studies, however, failed to indicate a synergistic antitumoral effect of the combination of EC-8042 with any of them (Figure S5). *In vivo* evaluation of the effect of EC-8042 in combination with docetaxel or carboplatin was also performed. As shown in Figure 6C, the combination of EC-8042 with docetaxel had a stronger antitumoral effect than the individual treatments, despite the lack of schedule optimization of the drug combination. In contrast, and in agreement with the *in vitro* data, no potentiation of the effect of carboplatin by EC-8042 was observed (Figure S6A). Body weights of animals treated with these drugs or their combination were not substantially modified by the treatments (Figure S6B).

## DISCUSSION

In this paper, we report the antitumoral action of the mithramycin analog EC-8042 in triple negative breast cancer. The rationale behind our study is double. On one hand is the recent identification that mithramycin shows an antitumoral action on TNBC cells [7]. On the other, the fact that mithramycin has a narrow therapeutic index moved us to test a mithralog selected by its lower toxicity and potentially higher therapeutic window [20].

EC-8042 exerted an antitumoral action both *in vitro* and *in vivo*. *In vitro* studies carried out in eight TNBC cell lines showed that the drug provoked a decrease in MTT metabolization values in a dose and time-dependent fashion. *In vivo*, the drug also caused a decrease in the growth of tumors developed from TNBC cells implanted in the mammary tissue of mice. Moreover, EC-8042 also favored the efficacy of taxanes *in vitro* and *in vivo*, and gemcitabine *in vitro*, two drugs currently used in the TNBC clinic. The effects in mice were observed without any significant effect of the drug on the weight of the mice, suggesting that the drug was well tolerated.

The mechanism of the antitumoral effect of EC-8042 was analyzed using flow cytometry as well as biochemical techniques. Treatment with the drug induced accumulation of cells with a diploid amount of DNA, indicating a blockade in G2 or M. Cell synchronization experiments using a double thymidine block, following by chasing of the cells in the absence or presence of the drug indicated that EC-8042 inhibited entry of cells in the M phase. Moreover, these experiments showed that cells treated with the drug evidenced a biochemical pattern compatible with G2 arrest. In fact, the drug caused accumulation of pCDK1-Y^15^. Phosphorylation of CDK1 at that residue inhibits its activity, therefore preventing cells from entering mitosis [24]. Such phosphorylation is controlled by the balance between the kinase Wee1 and the phosphatase Cdc25c [25]. Importantly, treatment with the drug strongly decreased the amount of Cdc25c, which is required for the dephosphorylation of pCDK1-Y^15^. The levels of Cdc25c are regulated by various transcription factors, including Sp1 [26], the putative cellular target of mithramycin and analogs [27]. In addition to strong down regulation of the levels of Cdc25c, the drug provoked accumulation of Wee1 which altogether could inactivate CDK1. These *in vitro* findings were coincident with the accumulation of Wee1 and pCDK1-Y^15^ in tumoral samples from mice xenografted with MDA-MB231 cells and treated with EC-8042.

In addition the effects of EC-8042 on the cell cycle, the drug also caused apoptosis, as indicated by accumulation of cells in the subG1 region of the histogram obtained by PI staining. However, the degree of apoptosis induced by the drug varied among different cell lines. In HBL100 cells, 500 nM of EC-8042 caused a strong induction of cell death, while in MDA-MB231 cells the effect was much lower. Evaluation of the mechanisms responsible for the cell death induced by the drug suggested that mitochondria could be involved in such process, as loss in MOMP was detected in both cell lines treated with the drug. Such loss in MOMP proportionally correlated with the degree of cell death induced by the drug. Interestingly, and even though clear differences were observed in MOMP loss in MDA-MB231 and HBL100 cells treated with 500 nM of EC-8042, biochemical analyses of caspase 3 and caspase 8 activation offered quantitatively similar results. Moreover, cleavage of PARP which is often used as readout of caspase 3 activity was induced by the drug in both cell lines. These data indicated that activation of caspase cascades were analogous in both cell lines and the differences in cell death induction by EC-8042 in the two cell lines could not be explained by the mere activation of caspases. In support of the latter were the experiments carried out using the pancaspase inhibitor Z-VAD-FMK. This compound only marginally inhibited cell death induced by EC-8042 in both cell lines, suggesting that large part of the proapoptotic action of the drug was due to activation of caspase-independent routes.

The biochemical characterization of the effects of EC-8042 on TNBC cells led to the identification of proteins whose levels could be used to define sensitivity/resistance to the action of the drug. Analyses of several proteins involved in G_2_ → M cell cycle progression and of SP1 indicated a certain degree of correlation among some of them and drug sensitivity. In fact, increased levels of Cdc25c and Sp1 appeared to confer resistance to the antitumoral action of EC-8042. It will be interesting to confirm such idea using genetic approaches to augment or decrease the levels of these proteins in TNBC cell models.

In summary, we show that the novel mithralog EC-8042 exerts antitumoral activity in TNBC. Moreover, EC-8042 behaved safely in mice, and augmented the action of some of the drugs commonly used in the therapy of TNBC. These biological properties of EC-8042 make this drug a worthy candidate for future development in the TNBC clinic.

## MATERIALS AND METHODS

### Reagents and antibodies

Cell culture media, fetal bovine serum (FBS) and tetramethylrhodamine ethyl ester (TMRE) were purchased from Invitrogen (Gaithersburg, MD). Propidium iodide (PI) and 3-(4,5-dimethylthiazol-2-yl)-2,5-diphenyltetrazolium bromide (MTT) were from Sigma Chemical (St Louis, MO,USA). Annexin V-FITC, Matrigel, Z-VAD-FMK, AC-IETD-AF and AC-DEVD-AFC were purchased from BD Biosciences (San Jose, CA). EC-8042 was provided by EntreChem SL (Oviedo, Spain) and obtained according to the procedure described before [20]. Carboplatin was from Pfizer (Madrid, Spain). Docetaxel was from Hospira UK Ltd (Warwickshire, United Kingdom). Vinorelbine was from Pierre Fabre (Barcelona, Spain). Gemcitabine was from Lilly (Madrid, Spain). BEZ235 and dasatinib were from LC Laboratories (Woburn, MA,USA). Other generic chemicals were purchased from Sigma-Aldrich (St. Louis, MO, USA), Roche Biochemicals, or Merck (Darmstadt, Germany). The anti-GAPDH, anti-cyclin E, anti-Wee1, anti-cyclin B, anti-CDK2, anti-CDK4, anti-p21, anti-MCL1, anti-BCL2, anti-Bax and anti-PARP antibodies were purchased from Santa Cruz Biotechnology (Santa Cruz, CA, USA). The anti-pCDK1 (Y^15^), anti-pH3, anti-p27, anti-Cdc25C, anti-Cdc25A, anti-SP1, anti-pRb^S807/811^, anti-pRb^S780^, anti-pRpb1, anti-Bid and anti-γH2Ax antibodies were from Cell Signalling Technologies (Beverly, MA, USA). The anti-cyclin A, anti-cyclin D1, anti-cyclin D3, anti-BUBR1, anti-BCLX, anti-APAF1, anti-XIAP and Anti-IAP1 antibodies were purchased from BD Biosciences (San Jose, CA, USA). The rabbit polyclonal anti-calnexin antibody was from Stressgen Biotechnologies Corporation (British Columbia, Canada). The anti-Bim and anti-Bak antibodies were from Calbiochem (La Jolla, CA, USA). Horseradish peroxidase conjugates of anti-rabbit and anti-mouse immunoglobulin G (IgG) were from Bio-Rad Laboratories (Hercules, CA, USA).

### Cell culture

All cell lines were cultured at 37°C in a humidified atmosphere in the presence of 5% CO_2_-95% air. Cells were grown in DMEM or in RPMI medium containing high glucose concentration (4,500 mg/liter) and antibiotics (penicillin at 100 mU/ml, streptomycin at 100 μg/ml) and supplemented with 10% FBS. Cell lines were provided by Drs. J. Losada and A. Balmain, (originally from Dr. J. W. Gray's Laboratory who in turn obtained them from the ATCC or from collections development in the laboratories of Drs. S. Ethier and A. Gazdar, to avoid errors occurring when obtained through “second-hand” sources) [28]. Cell identities were verified by STR analyses.

### Western blotting

MDA-MB231 and HBL100 cells were grown in DMEM with 10% of FBS and at 80% confluence were treated with EC-8042 for the times indicated. Cells were washed with phosphate-buffered saline (PBS) (NaCl, 137 mM; KCl, 2.7 mM; Na_2_HPO_4_, 8 mM; KH_2_PO_4_, 1.5 mM) and lysed in ice-cold lysis buffer (20 mM Tris-HCl, pH 7.0; NaCl, 140 mM; EDTA, 50 mM; 10% glycerol; 1% Nonidet P-40; 1 μM pepstatin; 1 μg/mL aprotinin; 1 μg/mL leupeptin; 1 mM phenylmethyl sulfonyl fluoride; 1 mM sodium orthovanadate). Lysates were centrifuged at 10,000 xg at 4°C for 10 minutes, and supernatants were transferred to new tubes. Samples were then boiled in electrophoresis sample buffer and run on SDS-PAGE gels at varying acrylamide concentrations, depending on the molecular weight of the proteins to be analyzed. After electrophoresis, the proteins in the gel were transferred to polyvinylidene difluoride membranes (PVDF) (Millipore Corporation, Bedford, MA, USA). Membranes were blocked in Tris-buffered saline with Tween (TBST) (100 mM Tris, pH 7.5; 150 mM NaCl; 0.05% Tween 20) containing 1% of bovine serum albumin (BSA) or 5% skimmed milk for 1-3 hours and then incubated with the corresponding antibody for 2-16 hours. After washing three times with TBST during 10 minutes, membranes were incubated with HRP-conjugated anti-mouse or anti-rabbit secondary antibodies for 30 minutes. After the secondary antibody, the membranes were washed three times with TBST and the bands were visualized by using enhanced chemiluminescence [29].

### Cell proliferation, cell cycle and apoptosis assays

Cells were plated in 24-well plates at 10,000-20,000 cells/well and cultured overnight in DMEM or RPMI + 10% FBS. The next day medium was replaced with DMEM or RPMI containing different concentrations of EC-8042. Cell proliferation was analyzed 48 and 72 hours later by an MTT-based assay as described [30]. Unless otherwise indicated, the results are presented as the mean ± standard deviation (SD) of quadruplicates of a representative experiment that was repeated at least three times.

To determine whether the combinations of EC-8042 with BEZ235, dasatinib, carboplatin, docetaxel, vinorelbine, or gemcitabine were synergistic, additive, or antagonistic we used the CalcuSyn v2.0 software program (Biosoft, Ferguson, MO) as described [31]. Results are plotted as the mean ±SD values of quadruplicates from two experiments.

For the analysis of the cell cycle profiles, cells were treated with EC-8042 during 48 hours and subsequently collected by pooling together the nonattached and attached cells. After washing with PBS, cells were fixed and permeabilized by ice-cold 70% ethanol overnight. Cells were centrifuged, resuspended in 500 μL of PBS containing 250 μg DNase-free RNAase A (Sigma-Aldrich) and incubated at room temperature for 2 hours. Then, 2.5 μg of propidium iodide (PI; Sigma-Aldrich) were added. DNA content and cell cycle analyses were performed using a BD Accuri C6 flow cytometer and the C6 software (BD Biosciences).

To analyze the effect of EC-8042 on the different phases of cell cycle, cells were synchronized in G1/S. To enrich cells in G1/S, cells were treated with thymidine (2 mM during 14 hours) as described [32]. Then the cells were treated with EC-8042 at different times and DNA content and cell cycle analyses were performed using a FACScalibur flow cytometer and the CellQuest software (BD Biosciences).

For apoptotic analyses, MDA-MB231 and HBL100 cells were treated with EC-8042 for 48 hours and subsequently collected by pooling together the nonattached and attached cells. Then, cells were washed with PBS and resuspended in 100 μL of binding buffer (10 mM HEPES/NaOH, pH 7.4; 140 mM NaCl; 2.5 mM CaCl_2_) containing 5 μL of Annexin V-fluorescein isothiocyanate (FITC; BD Biosciences) and 5 μL of 50 μg/mL PI. Cells were incubated for 15-30 minutes in the dark. After adding another 400 μL of binding buffer, labeled cells were analyzed in a BD Accuri C6 flow cytometer. Analyses of mitochondrial membrane potential in MDA-MB231 and HBL100 cells lines have been performed as described [33].

### Caspase activity assay

MDA-MB231 and HBL100 cells were lysed in apoptosis lysis buffer (20 mM Tris, 140 mM NaCl, 10 mM EDTA, 10% glycerol, 1% NP40, pH 7.0) supplemented with protease inhibitors. Protein concentration was determined by de BCA assay (Pierce) and 50 μg of cell lysates were placed in 96-well plates in triplicate. The final volume of the lysates was taken to to 100 μl by 1x Caspase buffer (25 mM HEPES pH 7.4, 150 mM NaCl, 1 mM EDTA, 0.1% CHAPS, 10% sucrose). One hundred μl of 2 x caspase reaction buffer (50 mM HEPES pH 7.4, 300 mM NaCl, 2 mM EDTA, 0.2% CHAPS, 20% sucrose, 20 mM DTT and 10 μM fluorescently labelled caspase substrate Ac-IETD-AFC or Ac-DEVD-AFC) was added to each well containing cell lysates. The plate was shaken to mix the solution and incubated at 37°C for 1 hour from light. Signals were measured at 400/505 nm in a fluorescent reader (BioTek).

### Xenograft studies

Mice were manipulated at the animal facility of the Centro de Investigación del Cáncer of Salamanca following legal guidelines. Female BALB/c nu/nu mice (7 weeks old) were obtained from Charles River Laboratories (Wilmington, MA, USA). A total of 5×10^6^ MDA-MB231 cells in 100 μl of DMEM and 100 μl of Matrigel (BD Biosciences) were injected subcutaneously into the right and left flank of each mouse. When tumours reached a mean volume of 100 mm^3^, animals (*n* = 32) were randomized into four groups (with equal average tumour volumes) (vehicle *n* = 8, EC-8042 *n* = 8, docetaxel *n* = 8, and EC-8042+docetaxel *n* = 8). Mice were treated every four days intravenously with 12 mg/kg of EC-8042, and weekly, intraperitoneally with docetaxel 15 mg/kg. Tumour diameters were serially measured by calliper twice per week and tumour volumes were calculated by the following formula: volume = width^2^ x length/2. Mice were sacrificed on day 41. For biochemical and drug accumulation analyses, tumor samples were obtained after sacrifice of the animals by CO_2_ inhalation, and 6 hours after being treated with the different drugs, and immediately frozen in liquid nitrogen. The tumors were minced, washed with PBS, and homogenized (Dispomix, L&M Biotech, Holly Springs, NC, USA) in ice-cold lysis buffer (1.5 ml/100 mg tumour). This homogenate was centrifuged at 10,000 xg for 20 minutes at 4°C, and the supernatants were transferred to new tubes.

### Statistical analyses

Comparisons of continuous variables between two groups for the mice tumor model experiments were performed using a two-sided Student's t-test. At least two independent experiments were performed for the *in vivo* studies. Differences were considered to be statistically significant when *P* values were less than 0.05. Statistical data are presented as the mean ± SD. Data were analyzed by the statistical software SPSS 21.0 (SPSS Inc., Chicago, IL).

## SUPPLEMENTARY MATERIAL FIGURES


