# Supplementary material for: Antitumoral activity of the mithralog EC-8042 in triple negative breast cancer linked to cell cycle arrest in G2
Source: Oncotarget. 2015 Sep 30;6(32):32856–67. doi: 10.18632/oncotarget.5942 (PMC4741734; doi:10.18632/oncotarget.5942)
Supplement: Supplementary file 1 [file oncotarget-06-32856-s001.pdf]

Antitumoral activity of the mithralog EC-8042 in triple negative breast cancer linked to cell cycle arrest in G2

Supplementary Material

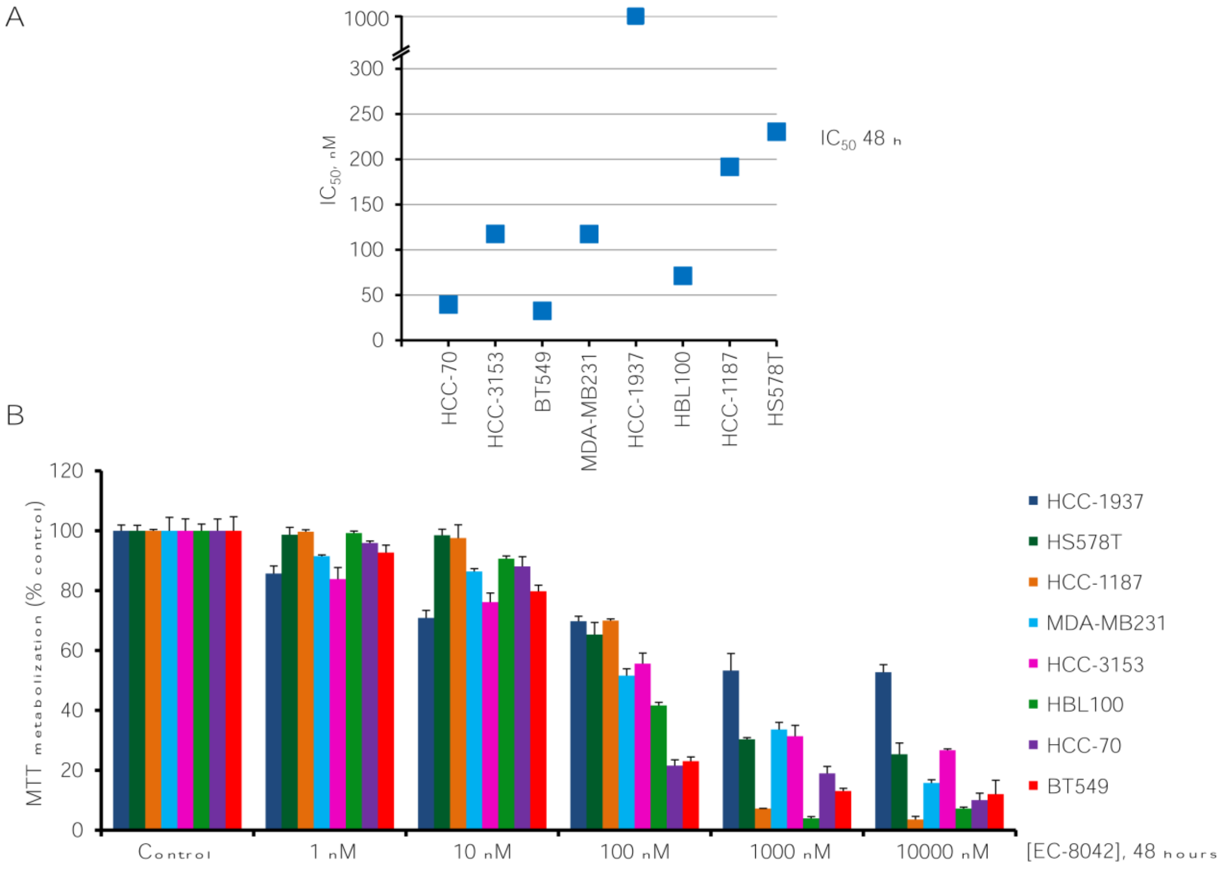

Figure S1. Antiproliferative effect of EC-8042 in triple negative breast cancer cell lines. (A) IC_{50} values obtained in the different TNBC cell lines after 48 hours of incubation with EC-8042. (B) Dose-response analyses of the effect of EC-8042 on the different TNBC cell lines treated with the drug for 48 hours. The data are plotted as the percentage of MTT metabolization with respect to control, vehicle-treated cells. Results are shown as the mean ± SD of quadruplicates of an experiment repeated three times.

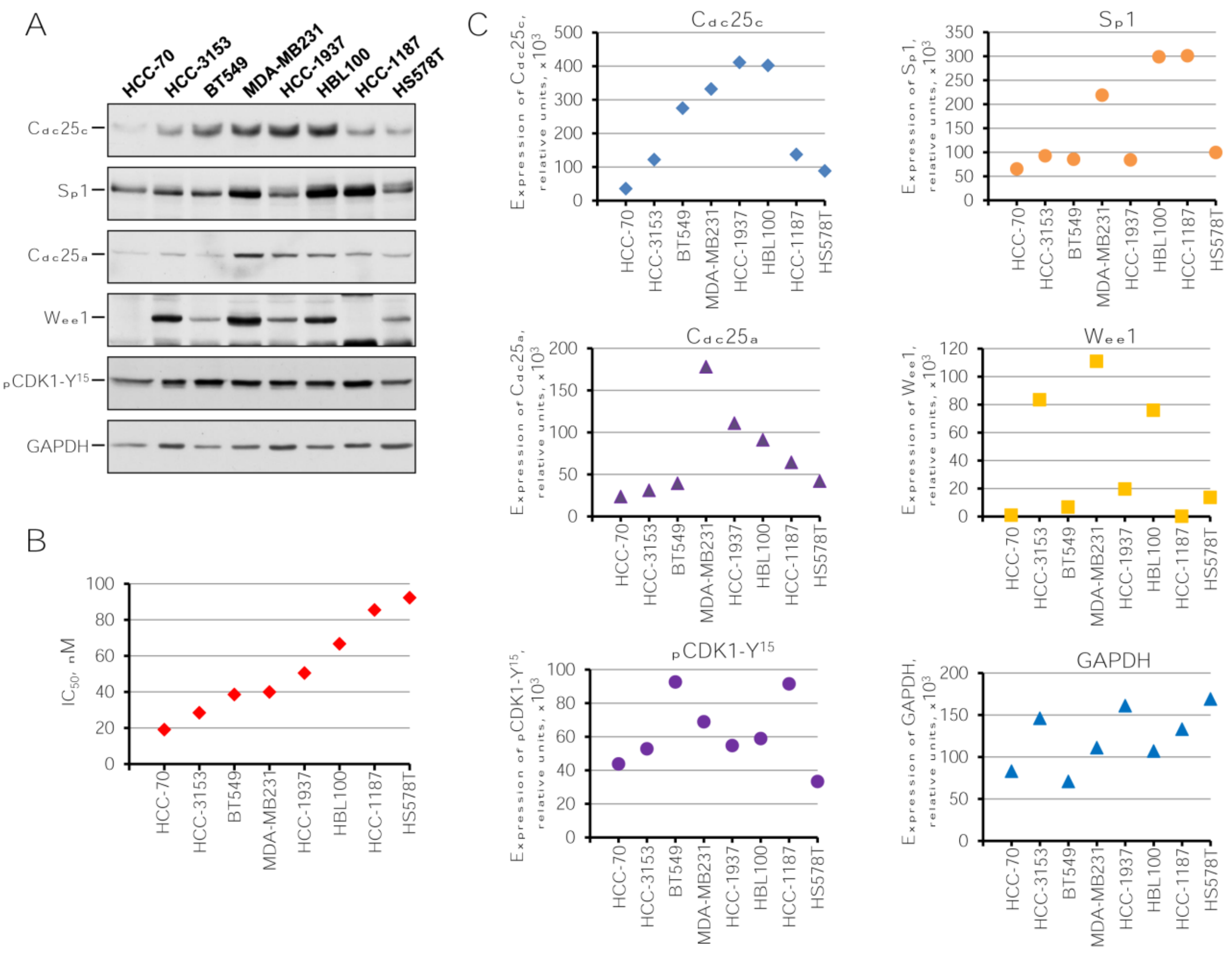

Figure S2. Expression of proteins involved in G2→M transition in TNBC cell lines. (A) Cells extracts of the different cell lines were prepared and Western blot analyses of the proteins indicated performed on the same amounts (50 μg) of protein. (B) Plot of the IC50 values obtained at 72 hours in the different TNBC cell lines. IC_{50} values are ordered from low (left) to high (right) values. (C) Quantitative analyses of the amount of the different proteins analyzed (in A), ordered with respect to their IC_{50} values.

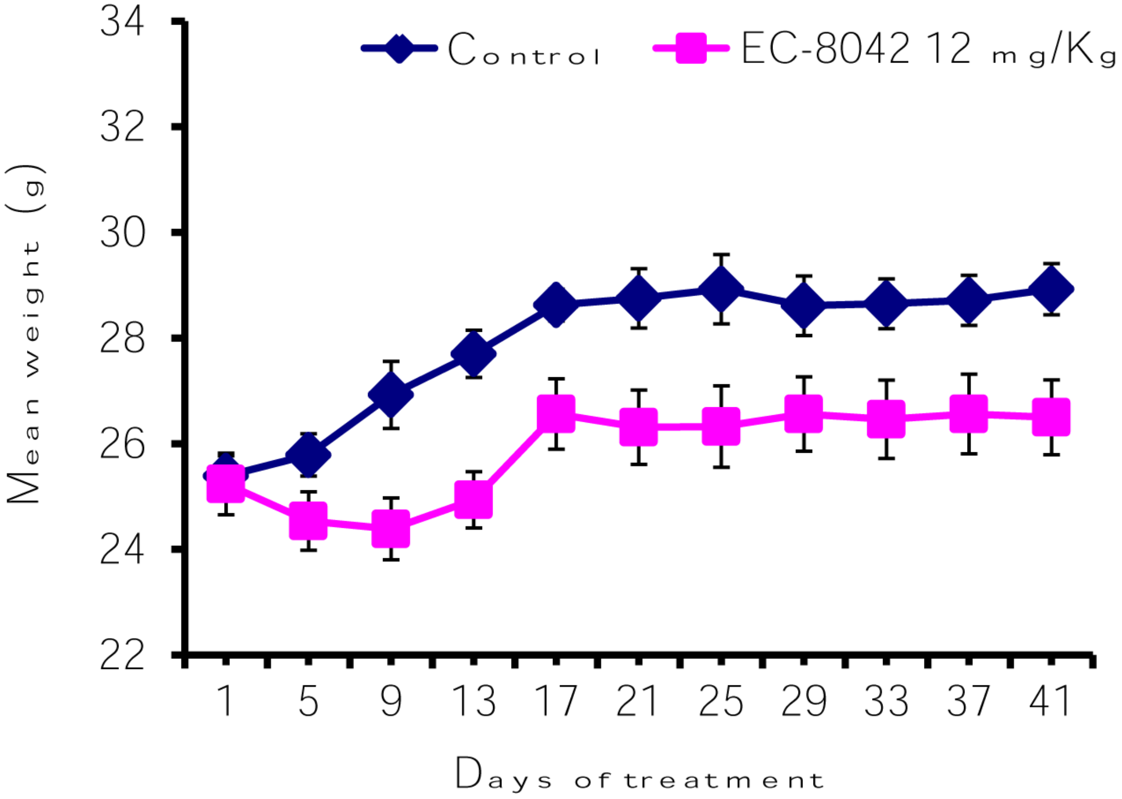

Figure S3. Effect of EC-8042 treatment on mice weight. MDA-MB231 cells were implanted in the mammary glands of female mice, and allowed to grow until tumors reached 100 mm^3. Mice were randomized to receive vehicle (control) or 12 mg/Kg EC-8042. Data are plotted as mean weights ± SD of eight mice/group.

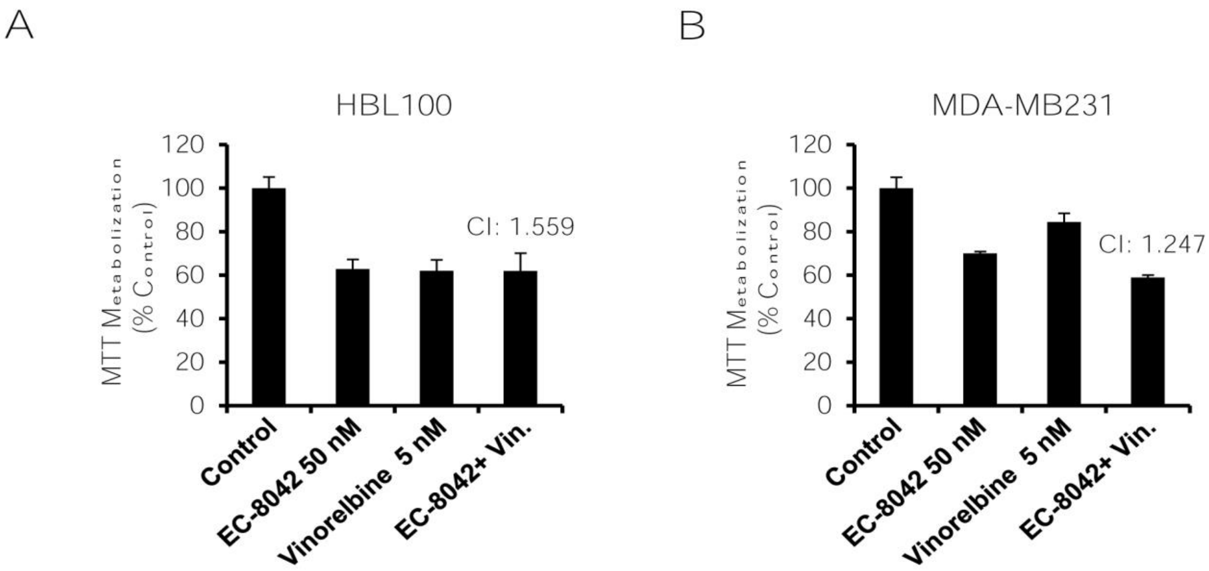

Figure S4. Effect of the combination of EC-8042 with vinorelbine. HBL100 (A) or MDA-MB231 (B) cells were treated for 48 hours with a fixed ratio model of combinations of EC-8042 with vinorelbine. MTT metabolization analyses were performed and data analyzed using the Chou-Talalay algorithm. The results shown in the figure correspond to only a single dose combination example in which synergy was observed as indicated by CI values below 1. The CI values are indicated for each of the combinations.

A

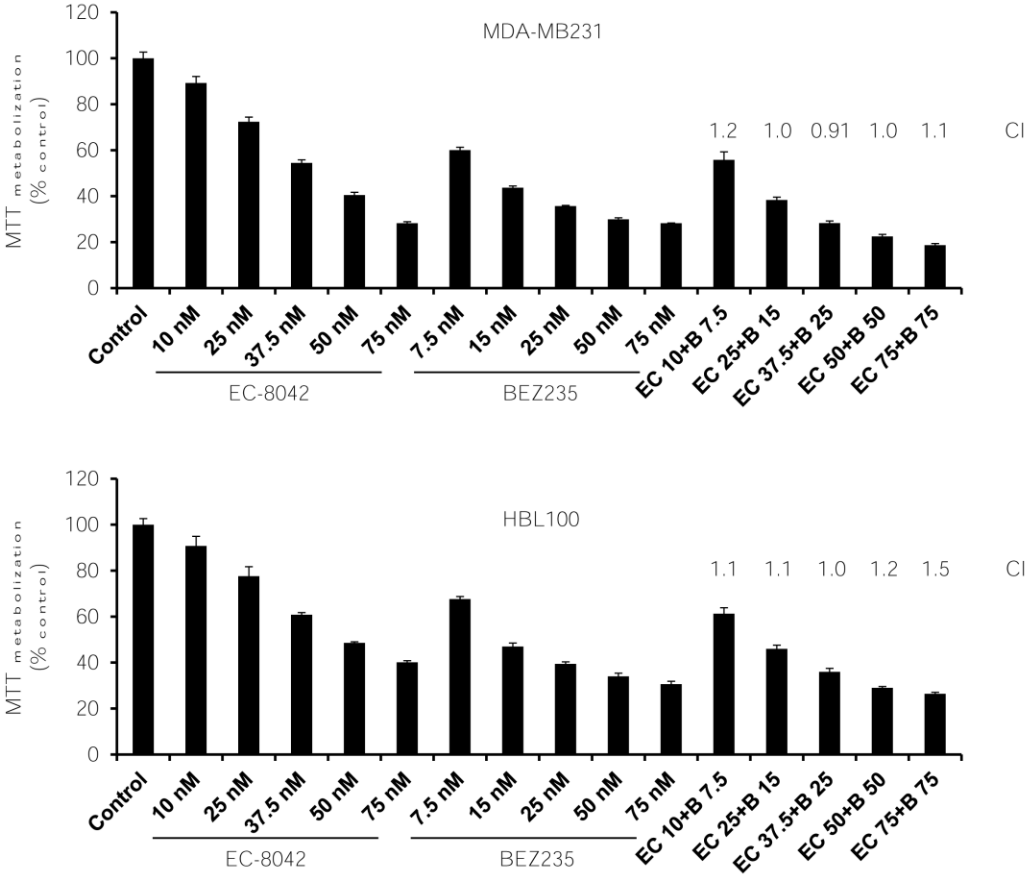
MDA-MB231
MTT metabolization (% control)
120
100
80
60
40
20
0
1.2 1.0 0.91 1.0 1.1 CI
Control
10 nM
25 nM
37.5 nM
50 nM
75 nM
7.5 nM
15 nM
25 nM
50 nM
75 nM
EC 10+B 7.5
EC 25+B 15
EC 37.5+B 25
EC 50+B 50
EC 75+B 75
EC-8042
BEZ235
HBL100
MTT metabolization (% control)
120
100
80
60
40
20
0
1.1 1.1 1.0 1.2 1.5 CI
Control
10 nM
25 nM
37.5 nM
50 nM
75 nM
7.5 nM
15 nM
25 nM
50 nM
75 nM
EC 10+B 7.5
EC 25+B 15
EC 37.5+B 25
EC 50+B 50
EC 75+B 75
EC-8042
BEZ235

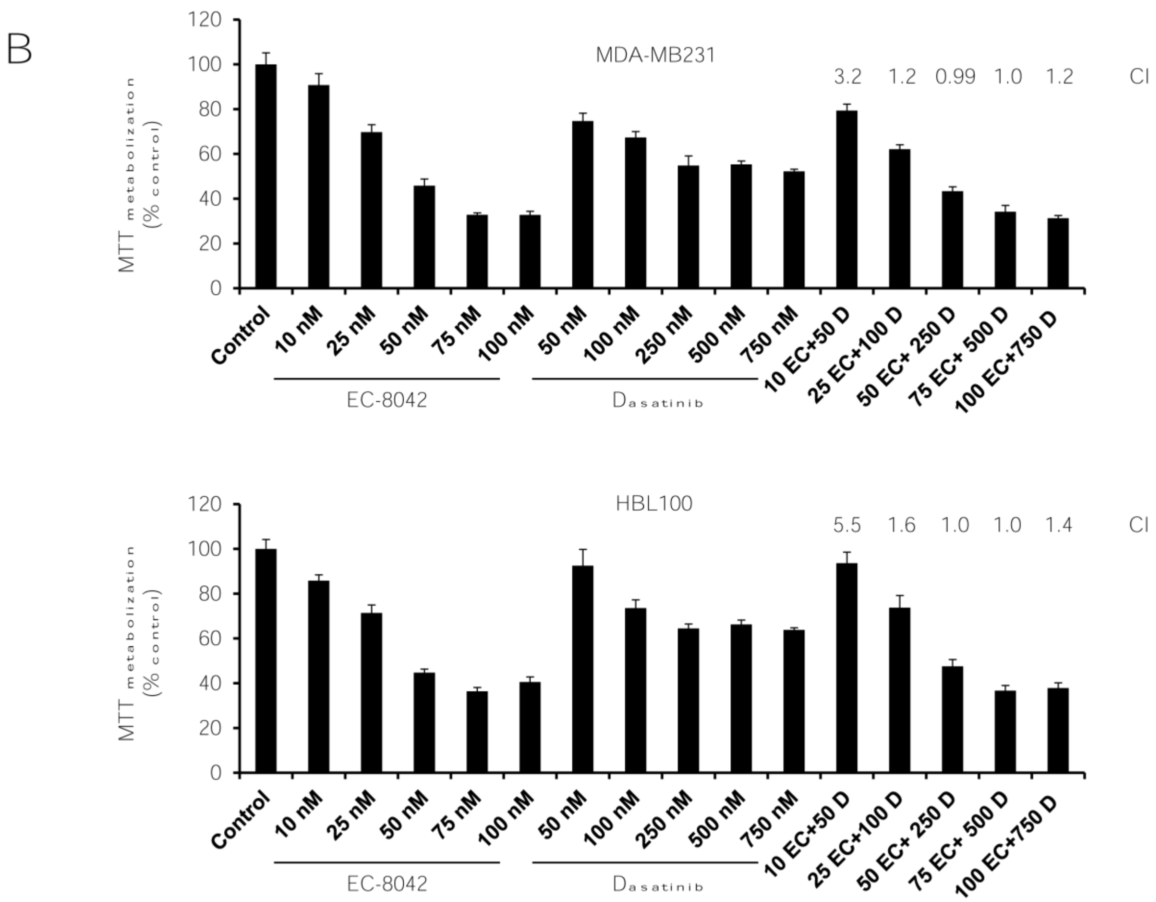

Figure S5. Effect of the combination of EC-8042 with BEZ235 or dasatinib. MDA-MB231 or HBL100 cells were treated for 48 hours with combinations of EC-8042 with BEZ235 (A) or dasatinib (B). MTT metabolization analyses were performed and data analyzed using the Chou-Talalay algorithm. The CI values are indicated for each of the combinations.

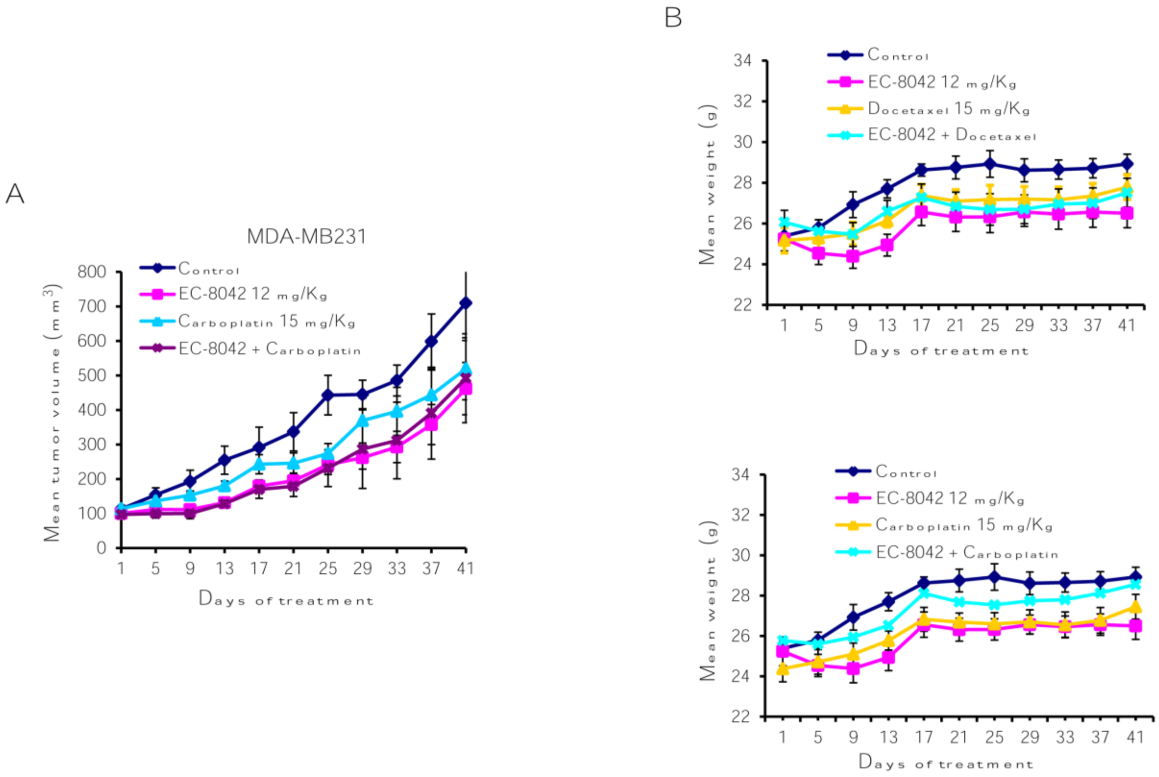

Figure S6. Antitumorigenic effect of EC-8042 in vivo in combination with carboplatin. (A) MDA-MB231 cells were implanted in the mammary glands of female mice, and allowed to grow until tumors reached 100 mm^3. Mice were randomized to receive the treatments indicated. Data are plotted as mean tumor volumes ± SD of eight mice/group. (B) Weighs of mice treated as indicated. Data are plotted as mean weights ± SD of eight mice/group.
